# Complications after sentinel node biopsy and inguinal lymph node dissection in penile cancer: a Danish national cohort study

**DOI:** 10.1007/s11255-026-05231-z

**Published:** 2026-06-11

**Authors:** J. Mølsted, A. B. Als, J. B. Jensen, M. Aagaard, J. K. Jakobsen

**Affiliations:** 1 Department of Clinical Medicine, Aarhus University, Aarhus, Denmark; 2 Department of Urology, Aarhus University Hospital, Aarhus, Denmark; 3 Department of Oncology, Aarhus University Hospital, Aarhus, Denmark; 4 Department of Urology, Copenhagen University Hospital, Copenhagen, Denmark

**Keywords:** Penile cancer, Lymph nodes, Sentinel node, Inguinal lymph node dissection, Complications

## Abstract

**Purpose:**

Sentinel node biopsy (SNB) and inguinal lymph node dissection (ILND) are central to penile cancer management, but ILND especially carries substantial morbidity. This nationwide study explores the extent and risk factors of postoperative complications after inguinal lymph node surgery in Danish penile cancer patients.

**Methods:**

We conducted a retrospective cohort study of penile cancer patients diagnosed in Denmark 2013–2025. Surgical procedures, pathological outcomes, and complications were extracted from medical records. Univariable and multivariable logistic regression with multiple imputation was used to assess predictors of complications.

**Results:**

Among 745 patients, 717 underwent 1348 SNB procedures and 184 underwent 241 ILND procedures. Postoperative morbidity increased substantially with procedural extent, with complications occurring after 9% of SNB procedures and after 31% of ILND procedures. Lymphoedema developed in 47% of patients following ILND versus 9% following SNB. Increasing lymph node yield independently predicted overall complications within both SNB and ILND cohorts and was additionally associated with wound infection following SNB. For lymphoedema, ILND carried five- to nine-fold higher odds compared with SNB, while higher BMI independently predicted lymphoedema risk (OR 1.07 per kg/m^2^, p = 0.002).

**Conclusion:**

Complications increase markedly with procedure extent. Increasing lymph node yield was associated with wound infection, while lymphoedema is primarily determined by procedure type. Poor predictive ability of patient characteristics limits pre-operative risk stratification.

**Supplementary Information:**

The online version contains supplementary material available at https://doi.org/10.1007/s11255-026-05231-z.

## Introduction

Accurate staging and management of regional lymph nodes are central to the prognosis of penile cancer, as the presence of inguinal nodal metastases remains the most important predictor of survival [1–4]. Early detection and appropriate treatment of micro-metastatic disease can be curative, whereas delayed intervention is associated with significantly worse oncological outcomes. Consequently, invasive nodal staging and treatment strategies play a pivotal role in contemporary management.

Dynamic sentinel node biopsy (SNB) and inguinal lymph node dissection (ILND) are important procedures for nodal assessment and treatment, but are primarily employed at high-volume tertiary referral centres. SNB is primarily used as a minimally invasive staging tool in clinically node-negative patients, aiming to identify occult metastases while reducing surgical morbidity. In contrast, ILND remains the standard therapeutic approach for patients with confirmed nodal disease or high-risk features, although it is associated with substantial morbidity.

The substantial morbidity associated with ILND in particular has important clinical implications beyond individual patient outcomes. High complication rates may deter clinicians from performing guideline-recommended nodal surgery, potentially compromising oncological outcomes in patients who would otherwise benefit from timely lymphadenectomy. This risk is particularly relevant in low-volume settings where limited surgical experience with inguinal lymph node surgery may compound procedural morbidity. Centralisation of penile cancer care to high-volume tertiary referral centres has been advocated as a strategy to mitigate this, by concentrating expertise and enabling standardisation of surgical technique and perioperative care [5]. Denmark has adopted a fully centralised model for penile cancer management, with all SNB and ILND procedures performed at two designated tertiary centres, which likely contributes to the procedural consistency reflected in the present cohort.

Despite their clinical benefit, both SNB and ILND procedures carry a risk of postoperative complications. Reported complication rates following SNB range from approximately 14–21% [6, 7], with most events being minor and related to wound healing or lymphatic leakage. In contrast, ILND is associated with considerably higher morbidity, with contemporary studies reporting complication rates between 19 and 58% [8–10], including wound infections, skin necrosis, lymphocele formation, and lymphoedema. These complications may lead to prolonged hospitalization, delayed adjuvant treatment, and reduced quality of life.

While complication profiles for SNB and ILND have been described separately, existing studies typically evaluate these procedures in isolation. This approach does not reflect real-world clinical pathways, where patients often undergo sequential or combined procedures, and where each inguinal basin may be exposed to different surgical interventions over time. As a result, the cumulative burden of complications at the patient and groin level remains poorly characterized.

Therefore, the aim of the present study is to evaluate the incidence and spectrum of complications following SNB and ILND in a national cohort of penile cancer patients, considering that many patients and individual groins undergo multiple procedures. By assessing complications in a combined and clinically representative framework, this study seeks to provide a more comprehensive understanding of the true morbidity associated with contemporary lymph node management in penile cancer.

## Patients and methods

### Study design and data source

This nationwide retrospective cohort study used data from the Danish National Penile Cancer database (DaPeCa-data) [4], which contains retrospectively collected clinical and pathological information on penile cancer patients managed at tertiary referral centres in Denmark since 2000. Data on postoperative complications are available from 2013 as limited access to records from early periods limits data validation possibilities.

Data on SNB and ILND were recorded at the inguinal basin level, allowing each patient to contribute up to two basin-level observations per procedure type. Only the initial SNB performed in each inguinal basin was included; subsequent SNB procedures performed for recurrent disease were excluded.

Patients lacking sufficient information for pathological staging or guideline-based risk stratification were classified as unstaged (e.g., pTx, pNx, cNx) and analyzed as a separate category. A non-curative treatment trajectory was defined as either palliative management or deviation from guideline-recommended treatment based on shared decision-making between patient and clinicians.

SNB was considered performed when at least one sentinel lymph node was excised from the specified inguinal basin. SNB positivity was defined as the presence of histologically confirmed metastatic involvement in any retrieved sentinel node.

ILND was undertaken in cases with documented nodal metastasis within an inguinal basin. Indications included a positive fine-needle aspiration, coarse needle biopsy, or positive SNB, as well as the presence of clinically or radiologically evident inguinal metastases. All ILND procedures in the cohort were performed as open surgery; the fascia-sparing approach was not recorded as a study variable.

Node counts were extracted from pathology reports in the medical records.

Since 2013, national Danish guidelines have recommended SNB in all inguinal basins in cN0 patients, irrespective of preoperative tracer uptake. In tracer-silent basins, repeat tracer injection on the same day is advised; alternatively, a limited inguinal dissection guided by blue dye and palpation may be performed with recovery of a few lymph nodes. These recommendations were introduced following evidence demonstrating that the previous strategy of leaving tracer-silent groins unexplored was associated with an unacceptable risk of missed metastatic disease [11, 12]. These cases were recorded as SNB procedures in this report.

In both Danish tertiary centres, SNB is occasionally performed in patients deemed unfit for completion ILND following shared decision-making. A negative result may provide reassurance and support classification as radically staged, while a positive result may reflect complete removal of limited metastatic disease or indicate residual nodal involvement that cannot be surgically addressed. These cases were recorded as SNB procedures and included in the present analysis.

In cases where nodal conglomerates prevented precise pathological node enumeration, for regression analyses these were coded as 10 nodes. Complication data were collected retrospectively from patient medical records; complications were not systematically recorded at the time of clinical care. Lower extremity oedema severity was reclassified as mild, moderate, or severe, based on the degree of patient-reported bother.

Complications during the first 90 postoperative days were graded according to the Clavien-Dindo (CD) classification [13] separately for each inguinal procedure. In cases where SNB was followed by ILND within 90 days, any complications occurring after the ILND procedure were attributed solely to the ILND procedure rather than the preceding SNB. Where a CD grade was assigned, complications were further subclassified into the following categories, with multiple answers permitted: ‘wound infection’, ‘lymphocele’, ‘bleeding/haematoma’, ‘systemic complications’, ‘deep venous thrombosis (DVT)’, and ‘other’. Skin problems were recorded as an additional category for ILND only. Lymphocele complications were recorded when a postoperative lymphocele resulted in unexpected clinical consequences, as the presence of a lymphocele after inguinal lymph node procedures is to be expected. Lymphoedema in this report covers chronic swelling of the lower limbs, and was recorded as a chronic patient-level sequela rather than an acute procedural complication, and was analysed at the patient level. Grade 5 complications were defined as death within 90 postoperative days unless a clearly unrelated cause was documented. Penile primary tumor surgery complications are not included in this study. In patients undergoing several procedures in one surgical setting, complications were attributed to a specific inguinal procedure when the site was clearly specified in the medical record (e.g. infection in a side-specific wound). Systemic complications and DVT were assigned to all procedures performed within the same operative episode preceding the event.

### Statistical analyses

All analyses were conducted using R version 4.5.2. Continuous variables were summarised using medians and interquartile ranges (IQRs), and categorical variables as counts and percentages.

The primary unit of analysis was the individual inguinal procedure. Analyses were conducted separately for the SNB and ILND cohorts.

Binary logistic regression was used to evaluate predictors of two primary outcomes: any postoperative complication (CD ≥ 1) and complications requiring treatment (CD ≥ 2). Univariable and multivariable models were fitted for each outcome within each cohort. Missing covariate data were handled using multiple imputation by chained equations (MICE) at patient level, generating 20 imputed datasets; imputed patient-level covariates were subsequently joined to the procedure-level analysis dataset. Regression estimates were pooled using Rubin’s Rules.

For secondary outcomes, binary logistic regression was performed for wound infection and lymphocele within each procedure cohort. Multivariable modelling was restricted to outcomes with at least 10 events per variable (EPV ≥ 10); outcomes below this threshold are reported with univariable results only and interpreted descriptively.

The following predictors were included in all models: age (continuous), smoking status (ever vs never), BMI (continuous), Charlson Comorbidity Index (continuous), ASA classification (binary: I-II vs III-V), inguinal tumour at diagnosis (binary), histologically verified metastatic pelvic lymph nodes (binary), procedure-specific lymph node yield (continuous), and extracted metastatic nodes at procedure (binary). Prior ipsilateral SNB was additionally included as a predictor in ILND models.

Inguinal tumour and presence of histologically verified pelvic lymph node metastases at diagnosis are patient-level variables despite being lateral characteristics. In patients undergoing bilateral procedures, the characteristics are duplicated across procedure rows in the procedure-level dataset. This applies equally to all patient-level covariates included in the models (age, BMI, smoking status, Charlson Comorbidity Index, ASA classification, and metastatic pelvic nodes); these variables are fixed patient characteristics carried forward to each procedure row and should be interpreted accordingly.

For the lymphoedema analyses, procedure group on patient level (SNB only, ILND only, SNB + ILND) was included as the primary exposure. To assess whether nodal burden independently predicts lymphoedema beyond procedure type, two alternative node-count variables were tested in separate models: total nodes removed across all procedures per patient, and maximum nodes removed from a single inguinal basin (summing SNB and ILND yield per side) per patient. Total number of inguinal procedures performed, with a maximum of one SNB and one ILND per inguinal basin, corresponding to a maximum of four procedures per patient, was included as a covariate in both node-count models.

## Results

### Cohort characteristics

Between January 1 st 2013 and December 31 st 2025, 872 patients with penile cancer were evaluated, of whom 745 underwent SNB, ILND, or both. A total of 1348 SNB procedures were performed in 717 patients, and 241 ILND procedures in 184 patients. Of the 745 patients, 561 underwent SNB only, 28 underwent ILND only, and 156 underwent both procedures. Baseline patient and tumour characteristics are presented in Table 1. Patients undergoing ILND had significantly more advanced and aggressive disease across all pathological variables compared with SNB-only patients. Patient-level characteristics (age, BMI, smoking status, comorbidity, and ASA classification) did not differ significantly between groups.

**Table 1:** Patient, tumour, and procedure characteristics by analysis cohort

Characteristic	Overall	SNB only	ILND (± SNB)	p-value^‡^
	n = 745	n = 561	n = 184	
Treatment strategy				
Curative	714 (96%)	544 (97%)	170 (92%)	0.013
Palliative	31 (4.2%)	17 (3.0%)	14 (7.6%)	
Age, years^†^	69 (61–77)	69 (60–77)	68 (61–76)	0.299
BMI, kg/m^2 †^	27.5 (24.8–30.6)	27.4 (24.5–30.4)	28.1 (25.1–31.2)	0.144
No data	66	56	10	
Smoking (ever vs never)				0.553
Ever	421 (57%)	311 (55%)	110 (60%)	
Never	285 (38%)	217 (39%)	68 (37%)	
No data	39 (5.2%)	33 (5.9%)	6 (3.3%)	
CCI category				0.415
Low risk (0–1)	128 (17%)	100 (18%)	28 (15%)	
Moderate risk (2)	132 (18%)	94 (17%)	38 (21%)	
High risk (3 +)	480 (64%)	363 (65%)	117 (64%)	
No data	5 (0.7%)	4 (0.7%)	1 (0.5%)	
ASA classification				0.145
ASA I-II	519 (70%)	400 (71%)	119 (65%)	
ASA III-V	219 (29%)	157 (28%)	62 (34%)	
No data	7 (0.9%)	4 (0.7%)	3 (1.6%)	
Pathological T-stage				< 0.001
pT1	347 (47%)	297 (53%)	50 (27%)	
pT2	283 (38%)	194 (35%)	89 (48%)	
pT3	105 (14%)	63 (11%)	42 (23%)	
pT4	7 (0.9%)	5 (0.9%)	2 (1.1%)	
pTx	3 (0.4%)	2 (0.4%)	1 (0.5%)	
Histological grade				< 0.001
G1	182 (24%)	156 (28%)	26 (14%)	
G2	262 (35%)	204 (36%)	58 (32%)	
G3	296 (40%)	197 (35%)	99 (54%)	
Gx	5 (0.7%)	4 (0.7%)	1 (0.5%)	
Vascular invasion	168 (23%)	103 (18%)	65 (35%)	< 0.001
No data	45 (6.0%)	32 (5.7%)	13 (7.1%)	
Perineural invasion	64 (8.6%)	41 (7.3%)	23 (13%)	0.036
No data	270 (36%)	203 (36%)	67 (36%)	
Pathological N-stage				< 0.001
pN0	504 (68%)	492 (88%)	12 (6.5%)	
pN1	87 (12%)	7 (1.2%)	80 (43%)	
pN2	27 (3.6%)	2 (0.4%)	25 (14%)	
pN3	123 (17%)	58 (10%)	65 (35%)	
pNx	4 (0.5%)	2 (0.4%)	2 (1.1%)	
Inguinal tumour at presentation	107 (14%)	46 (8.2%)	61 (33%)	< 0.001
No data	3 (0.4%)	3 (0.5%)	0 (0%)	
Metastatic pelvic nodes, histologically verified	22 (3.0%)	9 (1.6%)	13 (7.1%)	< 0.001
No data	58 (7.8%)	24 (4.3%)	34 (18%)	

*BMI* body mass index, *CCI* Charlson comorbidity index, *ASA* American society of anesthesiologists (ASA) physical status classification, *T-stage* primary tumor stage, *N-stage* nodal stage, *SNB* sentinel node biopsy, *ILND* inguinal lymph node dissection

^**†**^Median (Q1-Q3); n (%), ^**‡**^Wilcoxon rank sum test; Pearson’s Chi-squared test, comparing the ‘SNB only’ and ‘ILND (± SNB)’ groups

### Lymph node yield and procedure positivity

SNB procedures yielded 1–9 nodes, with 88% yielding one to three nodes and 12% more than three. ILND yielded 1–18 nodes, with 17% of dissections retrieving 1–3 nodes, 64% 4–10 nodes, and 16% yielded 11–18 nodes and 3.3% yielded conglomerates. Lymph node yield can be seen in Supplementary Table 1.

Of the 1348 SNB procedures, 230 (17%) yielded metastatic nodes. Of the 241 ILND procedures, 124 (51%) were positive overall. Among the 91 ILND procedures not preceded by ipsilateral SNB (performed predominantly for clinically or radiologically evident nodal disease) 86 (95%) were positive. Among the 150 completion ILND procedures performed following a positive SNB, 64 (43%) yielded additional metastatic nodes, indicating residual nodal disease beyond the sentinel node in nearly half of these cases.

### Patient and cancer characteristics according to complication status Table 2

**Table 2:** Cohort characteristics and complication rates

Characteristic	Overall	No complications	CD ≥ 1	p-value^‡^
	n = 1568	n = 1369	n = 199	
Age, years^†^	69 (61–77)	69 (60–77)	69 (61–78)	0.469
BMI, kg/m^2†^	27.7 (24.9–30.6)	27.7 (24.8–30.6)	28.1 (24.9–30.5)	0.470
No data	125	108	17	
Smoking (ever)				0.457
Ever	880 (56%)	761 (56%)	119 (60%)	
Never	615 (39%)	545 (40%)	70 (35%)	
No data	73 (4.7%)	63 (4.6%)	10 (5.0%)	
CCI category				0.598
Low risk (0–1)	266 (17%)	231 (17%)	35 (18%)	
Moderate risk (2)	281 (18%)	251 (18%)	30 (15%)	
High risk (3 +)	1017 (65%)	883 (64%)	134 (67%)	
No data	4 (0.3%)	4 (0.3%)	0 (0%)	
ASA classification				0.151
ASA I-II	1082 (69%)	953 (70%)	129 (65%)	
ASA III-V	476 (30%)	406 (30%)	70 (35%)	
No data	10 (0.6%)	10 (0.7%)	0 (0%)	
Inguinal tumour at diagnosis				0.001
Yes	218 (14%)	174 (13%)	44 (22%)	
No	1344 (86%)	1189 (87%)	155 (78%)	
No data	6 (0.4%)	6 (0.4%)	0 (0%)	
Histologically verified metastatic pelvic nodes				0.021
Yes	48 (3.1%)	36 (2.6%)	12 (6.0%)	
No	1386 (88%)	1219 (89%)	167 (84%)	
No data	134 (8.5%)	114 (8.3%)	20 (10%)	
Inguinal lymph nodes removed at procedure				< 0.001
1–3	1215 (77%)	1101 (80%)	114 (57%)	
4–5	175 (11%)	145 (11%)	30 (15%)	
> 5	178 (11%)	123 (9.0%)	55 (28%)	
Metastatic inguinal lymph nodes removed at procedure				0.003
0	1226 (78%)	1087 (79%)	139 (70%)	
≥ 1	342 (22%)	282 (21%)	60 (30%)	
Procedure type				< 0.001
SNB	1332 (85%)	1207 (88%)	125 (63%)	
ILND	236 (15%)	162 (12%)	74 (37%)	

*CD* Clavien Dindo classification of postoperatice complications, *BMI* body mass index, CCI Charlson Comorbidity Index, *SNB* sentinel node biopsy, *ILND* inguinal lymph node dissection

^†^Median (Q1-Q3); n (%), ^‡^Wilcoxon rank sum test; Pearson’s Chi-squared test

Across 1589 procedures, complication data were available for 1568, of whom 199 (13%) had at least one postoperative complication of CD grade ≥ 1. Patient characteristics (age, BMI, smoking, comorbidity, and ASA classification) did not differ significantly between procedures with and without complications. The presence of an inguinal tumour at diagnosis was more frequent in the complication group (22% vs 13%, p = 0.001), as were histologically verified metastatic pelvic nodes (6.0% vs 2.0%, p = 0.021). Procedures with complications involved greater nodal yield, with 28% retrieving more than five nodes compared with 9.0% in those without complications (p < 0.001), and a higher proportion had metastatic nodes removed (30% vs 21%, p = 0.003). ILND procedures accounted for 37% of all complications despite comprising only 15% of the total procedure volume.

### Complication rates by procedure type Table 3

**Table 3:** Complication rates by procedure type and SNB-ILND timing

Characteristic	Overall	SNB	ILND overall	ILND (+ SNB ≤ 30 d)	ILND (+ SNB > 30 d)	ILND only	p-value^†^
Procedures	n = 1589	n = 1348	n = 241	n = 58	n = 92	n = 91	
Patients	n = 745	n = 561	n = 184	n = 49	n = 72	n = 26	
Any complication (CD ≥ 1)	199 (13%)	125 (9.4%)	74 (31%)	15 (26%)	29 (32%)	30 (34%)	< 0.001
Complication CD ≥ 2	171 (11%)	109 (8.2%)	62 (26%)	13 (23%)	24 (27%)	25 (28%)	< 0.001
Clavien-Dindo grade							< 0.001
Grade 0	1369 (87%)	1207 (91%)	162 (69%)	42 (74%)	61 (68%)	59 (66%)	
Grade 1–2	82 (5.2%)	55 (4.1%)	27 (11%)	7 (12%)	7 (7.8%)	13 (15%)	
Grade 3a-3b	109 (7.0%)	67 (5.0%)	42 (18%)	8 (14%)	18 (20%)	16 (18%)	
Grade 4a-4b	6 (0.4%)	3 (0.2%)	3 (1.3%)	-	2 (2.2%)	1 (1.1%)	
Grade 5	2 (0.1%)	-	2 (0.8%)	-	2 (2.2%)	-	
No CD data	21	16	5	1	2	2	
Wound infection	117 (7.4%)	82 (6.1%)	35 (15%)	8 (14%)	10 (11%)	17 (19%)	< 0.001
Lymphocele	71 (4.5%)	37 (2.7%)	34 (14%)	7 (12%)	14 (15%)	13 (14%)	< 0.001
Bleeding/haematoma	7 (0.4%)	5 (0.4%)	2 (0.8%)	-	1 (1.1%)	1 (1.1%)	0.300
Systemic complication	14 (0.9%)	7 (0.5%)	7 (2.9%)	-	6 (6.5%)	1 (1.1%)	< 0.001
DVT/thrombo-embolic event	8 (0.5%)	6 (0.4%)	2 (0.8%)	1 (1.7%)	-	1 (1.1%)	0.200
Other	22 (1.4%)	12 (0.9%)	10 (4.1%)	2 (3.4%)	5 (5.4%)	3 (3.3%)	< 0.001
Skin problems	3 (0.2%)	-	3 (1.2%)	-	2 (2.2%)	1 (1.1%)	0.003
Lymphoedema (patient level)	132 (18%)	49 (8.8%)	83 (47%)	22 (45%)	35 (49%)	12 (46%)	< 0.001

n = number of procedures

n (%); percentages calculated from non-missing observations

*CD* Clavien-Dindo classification, *SNB* sentinel node biopsy, *ILND* inguinal lymph node dissection. ILND only ILND performed without prior ipsilateral SNB. *ILND (*+ *SNB* ≤ * 30 d)* ILND with prior ipsilateral SNB less than 30 days before, *ILND (*+ *SNB* > * 30 d)* ILND with prior ipsilateral SNB more than 30 days before

^†^ Fisher’s Exact Test

**Fig. 1:**
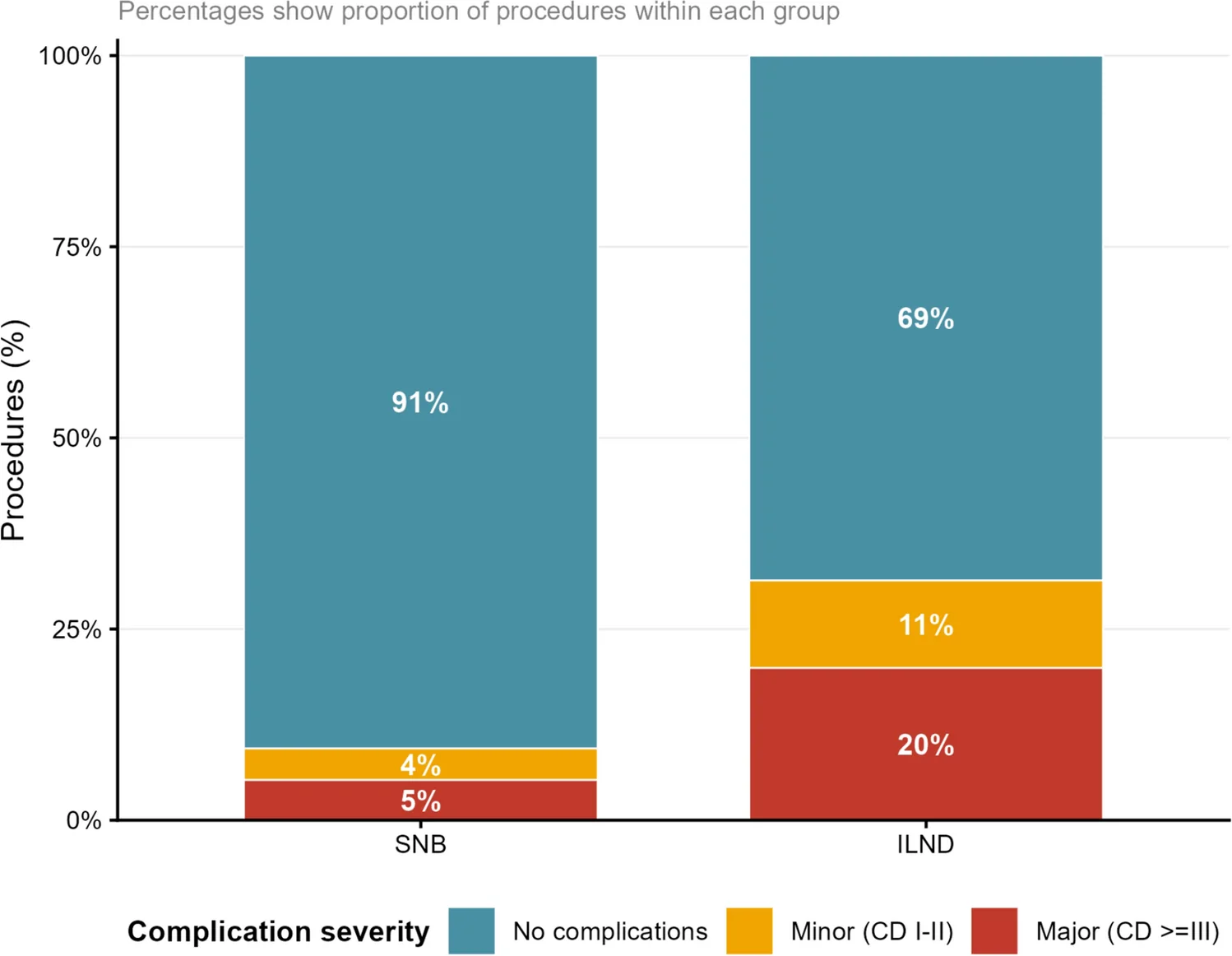
Complication severity by procedure type. Stacked bar chart showing the proportion of patients experiencing no complications (Clavien-Dindo grade 0, blue), minor complications (Clavien-Dindo grades I-II, orange), and major complications (Clavien-Dindo grade ≥ III, red) across three procedure groups. Percentages shown within each segment represent the proportion of patients in that procedure group experiencing each severity level. *CD* Clavien-Dindo classification, *ILND* inguinal lymph node dissection, *SNB* sentinel node biopsy

Complication rates differed markedly between SNB and ILND (p < 0.001). See Fig. 1.

Among 1348 SNB procedures, the overall complication burden was modest, as a total of 125 procedures (9.4%) were assigned a CD ≥ 1 grade and 109 (8.2%) a CD ≥ 2 grade. Wound infection was the dominant complication type, arising after 82 procedures (6.1%), with lymphocele recorded after 37 (2.7%). Systemic complications, DVT, and bleeding were each rare, occurring after 7 (0.5%), 6 (0.4%), and 5 (0.4%) procedures respectively. The majority of complications were minor: Grade 1–2 events accounted for 55 procedures (4.1%) and Grade 3a-3b for 67 (5.0%). Grade 4a-4b events were exceptional (n = 3, 0.2%) and no deaths within 90 days were recorded following SNB.

Postoperative complications were more prevalent after ILND. Of 241 ILND procedures, 74 (31%) resulted in a CD ≥ 1 complication and 62 (26%) in a CD ≥ 2 complication. Wound infection and lymphocele were approximately equally common after 35 (15%) and 34 (14%) procedures respectively. Systemic complications arose after 7 procedures (2.9%), skin problems after 3 (1.2%), and bleeding and DVT after 2 procedures each (0.8%). The severity distribution also shifted considerably: Grade 3a-3b events occurred after 42 ILND procedures (18%), Grade 4a-4b after 3 (1.3%), and two deaths were recorded after ILND. When stratified by prior ipsilateral SNB, the complication rates were similar across ILND subgroups: 34% in ILND procedures not preceded by an SNB procedure (n = 91), 26% in ILND preceded by ipsilateral SNB within 30 days (n = 58), and 32% when the preceding SNB was performed more than 30 days earlier (n = 92).

Lymphoedema, recorded at the patient level as a chronic sequela rather than an acute procedural complication, was strikingly more prevalent after ILND. It affected 83 of 184 ILND patients (45%) compared with 49 of 561 SNB-only patients (8.8%, p < 0.001). Rates were consistent across ILND subgroups regardless of prior ipsilateral SNB procedure.

### Primary outcomes: risk factors for Clavien-Dindo-graded complications

In the SNB cohort, lymph node yield was the only predictor associated with overall complications in both univariable and multivariable analysis. Each additional node removed was independently associated with higher odds of CD ≥ 1 (multivariable OR 1.14, 95% CI 1.01–1.30, p = 0.039) and CD ≥ 2 (OR 1.18, 95% CI 1.03–1.35, p = 0.016). No patient-level characteristic reached significance in either univariable or multivariable analysis for either primary outcome in the SNB cohort.

**Fig. 2:**
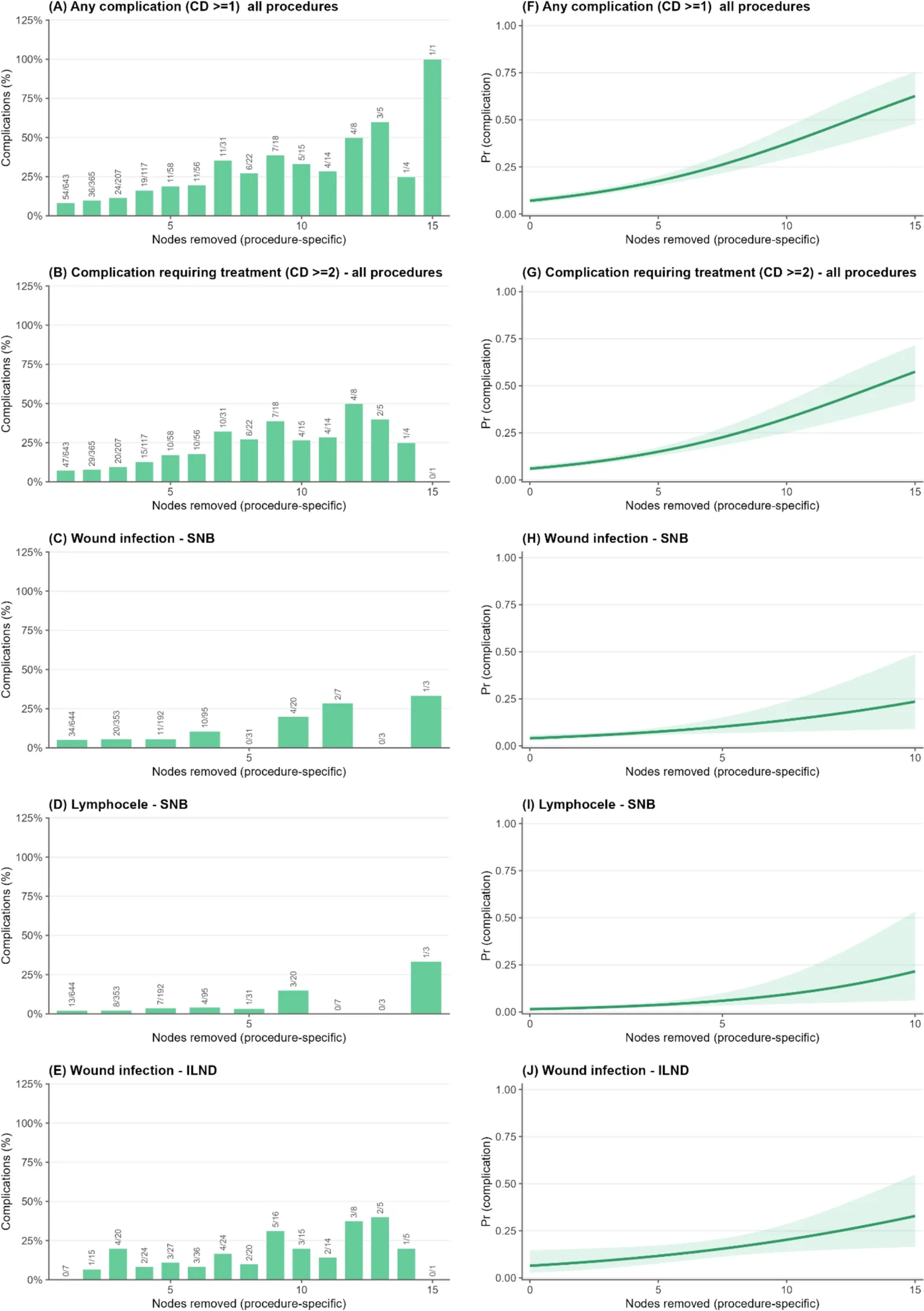
Association between lymph node yield and complication risk following inguinal nodal surgery for penile cancer. Bar graphs **A**-**E** show observed complication rates at each lymph node count, with fractions above bars indicating the number of events over the total number of groins at that yield. Logistic regression curves. **F**-**J** show the predicted probability of each complication as a function of lymph node yield, with shaded areas representing 95% confidence intervals. **A**, **F** Any complication (CD ≥ 1) in the full cohort. **B**, **G** Complication requiring intervention (CD ≥ 2) in the full cohort. **C**, **H** Wound infection following SNB. **D**, **I** Lymphocele formation following SNB. **E**, **J** Wound infection following ILND. *CD* Clavien-Dindo classification, *ILND* Inguinal lymph node dissection, *OR* odds ratio, *SNB* sentinel node biopsy

In the ILND cohort, lymph node yield was similarly the sole independent predictor of overall complications. Each additional node removed was associated with higher odds of CD ≥ 1 (multivariable OR 1.10, 95% CI 1.00–1.20, p = 0.039) and CD ≥ 2 (OR 1.12, 95% CI 1.02–1.23, p = 0.015). In univariable analysis, CCI approached significance for CD ≥ 1 (OR 1.14, 95% CI 0.99–1.32, p = 0.065), and metastatic pelvic nodes showed a clinically but not statistically significant association with CD ≥ 1 (OR 2.30, 95% CI 0.89–5.95, p = 0.086) and CD ≥ 2 (OR 2.41, 95% CI 0.93–6.22, p = 0.070). Prior ipsilateral SNB was not associated with complication risk in either primary ILND outcome in univariable or multivariable analysis (Fig. 2A, B, F, G).

### Secondary outcomes-risk factors for specific complication types

*SNB—wound infection*: Lymph node yield was the only independent predictor of wound infection after SNB (multivariable OR 1.22 per node, 95% CI 1.05–1.41, p = 0.008) (Fig. 2C, H).

*SNB—lymphocele*: In univariable analysis, lymph node yield was the strongest predictor (OR 1.34 per node, 95% CI 1.11–1.62, p = 0.002). Smoking showed an elevated but non-significant association (OR 1.94, 95% CI 0.93–4.05, p = 0.077)) (Fig. 2D, I).

*ILND—wound infection*: In univariable analysis, no predictor reached statistical significance. Lymph node yield showed the strongest association (OR 1.10, 95% CI 0.99–1.22, p = 0.066) (Fig. 2E, J).

*ILND—lymphocele*: In univariable analysis, the presence of metastatic pelvic nodes showed the strongest association (OR 4.64, 95% CI 1.80–11.94, p = 0.002), followed by inguinal tumour at diagnosis (OR 2.35, 95% CI 1.12–4.91, p = 0.023) and CCI (OR 1.23, 95% CI 1.03–1.47, p = 0.021).

*Lymphoedema*: Procedure type was the dominant independent predictor of lymphoedema. Compared with SNB-only patients, those undergoing both SNB and ILND had nearly nine times higher odds of lymphoedema (multivariable OR 8.78, 95% CI 5.56–13.85, p < 0.001), and those undergoing ILND only had nearly seven times higher odds (OR 6.84, 95% CI 2.70–17.30, p < 0.001). Among patient-level factors, higher BMI was the only independent predictor (OR 1.07 per kg/m^2^, 95% CI 1.03–1.12, p = 0.002).

**Fig. 3:**
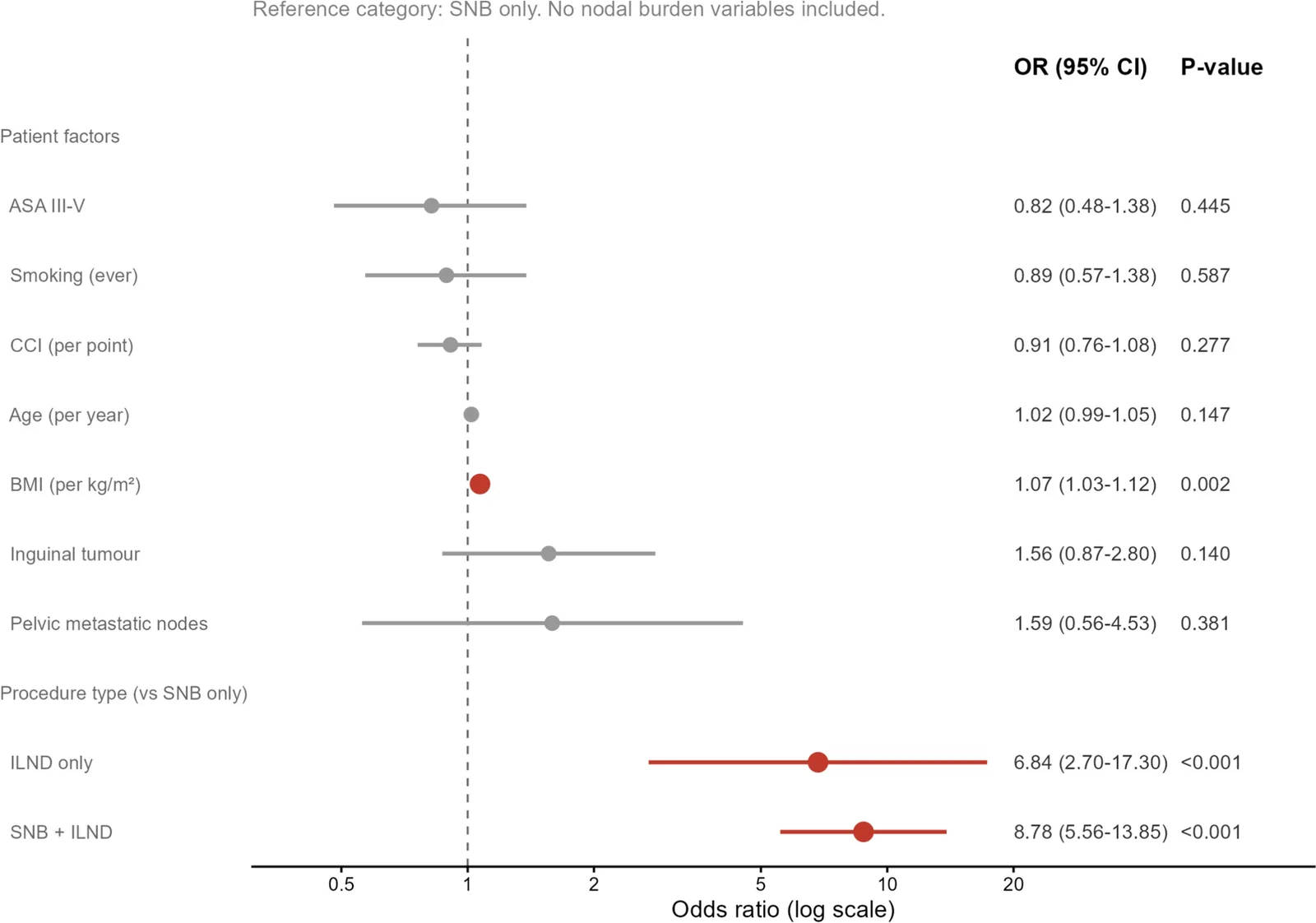
Multivariable predictors of lymphoedema. Forest plot showing adjusted odds ratios with 95% confidence intervals from multivariable logistic regression analysis in the full cohort (n = 745). Reference category for procedure type is SNB-only. Red points indicate statistically significant predictors (p < 0.05); grey points indicate non-significant associations. Total procedures: accumulated SNB and ILND procedures with a max of 2 of each procedure per patient, Total nodes removed: the total amount of removed inguinal lymph nodes per patient from SNB and ILND procedures. *BMI* body mass index; *CCI* Charlson Comorbidity Index, *CI* confidence interval, *ILND* inguinal lymph node dissection, *OR* odds ratio, *SNB* sentinel node biopsy

To further assess the independent contribution of nodal burden to lymphoedema risk, three additional variables were entered alongside procedure group in separate models: total nodes removed across all procedures and groins per patient, maximum nodes removed from a single groin per patient, and total number of inguinal procedures per patient. In univariable analysis, all three variables were significantly associated with a higher odds of lymphoedema: total nodes removed (OR 1.18, 95% CI 1.12–1.24, p < 0.001), maximum unilateral nodes (OR 1.27, 95% CI 1.19–1.36, p < 0.001), and total procedures (OR 2.18, 95% CI 1.72–2.76, p < 0.001). In multivariable analysis, none of the three reached statistical significance when adjusted for procedure group (total nodes OR 1.03, 95% CI 0.98–1.09, p = 0.279; maximum unilateral nodes OR 1.05, 95% CI 0.97–1.14, p = 0.265; total procedures OR 0.87, 95% CI 0.52–1.47, p = 0.700) (Fig. 3).

Due to a limited number of events not reaching the EPV of 10 required for modelling, multivariable modelling was not performed for SNB lymphocele, ILND wound infection and ILND lymphocele.

All regression analysis results can be seen in Supplementary Table 2.

## Discussion

In this nationwide cohort study of penile cancer patients undergoing inguinal nodal surgery the overall complication rates observed were broadly consistent with contemporary literature for both SNB and ILND [6, 7, 10, 14, 15]. Consistent with previous literature, ILND carried substantially greater morbidity than SNB. Approximately one-third of ILND procedures resulted in postoperative complications, compared with fewer than 10% of SNB procedures. Higher BMI independently predicted lymphoedema, consistent with previous reports identifying obesity as an important contributor to postoperative morbidity after ILND [10]. Together, these findings underscore the substantial chronic morbidity associated with ILND and support continued use of SNB as the preferred minimally invasive staging approach in eligible cN0 patients.

An important finding of this study was that higher lymph node yield independently predicted overall complications within both the SNB and ILND cohorts and was additionally associated with wound infection following SNB.

Nevertheless, lymph node yield should be interpreted cautiously and not necessarily as a directly modifiable causal factor. Higher node counts likely reflect several overlapping aspects of operative extent and complexity, including broader tissue dissection, anatomical variation, pathological processing techniques, surgeon preference, and underlying nodal disease burden. Thus, lymph node yield may function both as a quantitative marker of surgical extent and as a surrogate for procedural complexity.

Only 2.4% of SNB procedures resulted in retrieval of six or more lymph nodes. From a conceptual perspective, removal of numerous nodes during a procedure intended as a SNB may begin to resemble a limited lymphadenectomy, thereby reducing some of the minimally invasive benefit inherent to the sentinel node approach. However, no evidence-based consensus exists regarding an “optimal” SNB node yield [16, 17] and large heterogeneity exists across penile cancer centres in how lymph node procedures are handled [18]. Furthermore, the final lymph node count depends not only on surgical retrieval but also on pathological assessment and specimen processing, which may influence the number of identified nodes and limit the validity of rigid threshold-based interpretations [19–22].

The substantial morbidity associated with ILND also highlights the continued clinical importance of treatment de-escalation strategies. Such paradigms have transformed axillary management in breast cancer, where selected patients with limited sentinel-node metastases can safely omit completion lymphadenectomy without compromising oncological outcomes. Direct comparison with penile cancer must, however, be made cautiously given the biological and epidemiological differences between the diseases.

Several methodological considerations warrant discussion. Complication ascertainment was retrospective and based on review of medical records rather than prospectively standardised reporting. Minor complications managed conservatively or outside tertiary referral centres may therefore have been underreported, potentially leading to underestimation of the true morbidity burden, particularly for low-grade events. The absence of diabetes status as a standalone variable represents an important limitation. Diabetes is a recognized risk factor for complications after ILND, with reported odds ratios of approximately 3 in prior series by Likitpanpisit and Siriboonrid [15], though its role after SNB remains unclear [7].

For SNB procedures followed by ILND within 90 days, the postoperative observation period for Clavien-Dindo complication registration was truncated at the time of ILND. This introduces a potential risk of underreporting complications in the SNB cohort. As only 11% of SNB procedures were followed by ILND, the magnitude of this bias is likely small. Conversely, some complications attributed to ILND may have originated from a preceding SNB procedure, although the impact of this potential attribution bias is likely limited given the substantially lower morbidity associated with SNB.

The study possesses several important strengths. The nationwide design and centralised management of penile cancer in Denmark minimised referral bias and enabled inclusion of a large contemporary cohort with detailed clinical and pathological data. Furthermore, the high national volume and centralisation of SNB procedures likely contributed to procedural consistency and strengthens the validity and generalisability of the findings. Finally, direct extraction of data from patient medical records rather than registries ensured near-complete capture of procedures and complications, further strengthening internal validity.

The optimal management of inguinal lymph node metastases in penile cancer remains an active area of investigation. Although ILND remains essential for oncological control in patients with inguinal nodal metastases, the substantial morbidity associated with the procedure highlights the importance of treatment de-escalation strategies. As demonstrated in the present study, 57% of completion ILND procedures yielded no additional metastatic nodes. While this finding can only be appreciated retrospectively, it highlights that many patients are exposed to the morbidity of completion ILND despite having no residual nodal disease beyond that identified at SNB. In a large multicentre study, de Vries et al. investigated clinicopathological predictors of additional inguinal nodal metastases in sentinel node-positive patients with the aim of identifying individuals who might safely avoid completion ILND [23]. However, sufficiently robust predictors for clinical decision-making remain lacking, and completion ILND therefore remains the standard of care for sentinel node-positive disease. Concurrently, efforts to reduce treatment-related morbidity have stimulated interest in less invasive surgical approaches. Robot-assisted ILND is increasingly adopted in specialised centres and may reduce postoperative complications, although operative times are generally longer than with open surgery [24, 25]. Other minimally invasive approaches have also shown promising results [26, 27], but the available evidence is largely limited to small institutional series, reflecting both the rarity of penile cancer and the highly specialised nature of inguinal nodal surgery.

## Conclusion

This nationwide study confirms the substantially higher morbidity associated with ILND compared with SNB in penile cancer patients undergoing inguinal nodal surgery. Increasing lymph node yield was consistently associated with postoperative complication risk, suggesting that procedural extent and surgical disruption are important drivers of morbidity. These findings support continued adherence to minimally invasive principles during nodal staging and highlight the need for ongoing efforts toward treatment de-escalation.

## Supplementary Information

Below is the link to the electronic supplementary material.

Supplementary file1 (DOCX 42 KB)

Supplementary file2 (DOCX 47 KB)

## Data Availability

The data that support the findings of this study are not publicly available due to Danish data protection legislation and regulations governing access to national health registry data. Data are available from the authors upon reasonable request and with permission from the relevant Danish authorities.
