# Supplementary material for: Complications after sentinel node biopsy and inguinal lymph node dissection in penile cancer: a Danish national cohort study
Source: Int Urol Nephrol. 2026 Jun 11;58(11):4799–810. doi: 10.1007/s11255-026-05231-z (PMC13694304; doi:10.1007/s11255-026-05231-z)
Supplement: Supplementary file 1 [file 11255_2026_5231_MOESM1_ESM.docx]

| **Supplementary table 1**  **Nodes removed SNB and ILND procedures** | | | |
| --- | --- | --- | --- |
| **SNB** | | **ILND** | |
| **Characteristic** | **n = 1,348** | **Characteristic** | **n = 241** |
| **Nodes removed at SNB** |  | **Nodes removed at ILND** |  |
| 0 | 0 (0%) | 0 | 0 (0%) |
| 1-3 | 1,189 (88%) | 1-3 | 42 (17%) |
| 4-5 | 126 (9.3%) | 4-10 | 154 (64%) |
| 6+ | 33 (2.4%) | 11-16 | 35 (15%) |
| Conglomerate | 0 (0%) | 17+ | 2 (0.8%) |
|  |  | Conglomerate | 8 (3.3%) |
| **Positive nodes at SNB** |  | **Positive nodes at ILND** |  |
| 0 | 1,117 (83%) | 0 | 117 (49%) |
| 1 | 180 (13%) | 1-3 | 107 (44%) |
| 2 | 42 (3.1%) | 4-7 | 8 (3.3%) |
| 3 | 5 (0.4%) | 8-12 | 2 (0.8%) |
| 4+ | 3 (0.2%) | 13+ | 0 (0%) |
| Conglomerate | 0 (0%) | Conglomerate | 7 (2.9%) |
| No data | 1 |  |  |
| **Nodes removed** | Mean^†^ 1.99 (1.29) / Median 2.00 (1.00-3.00) | **Nodes removed** | Mean^†^ 7 (3) / Median 6 (4-9) |
| **Positive nodes** | Mean^†^ 0.22 (0.54) / Median 0.00 (0.00-0.00) | **Positive nodes** | Mean^†^ 1 (1) / Median 1 (0-1) |
| No data | 1 |  |  |
| SNB: Sentinel node biopsy, ILND: Inguinal lymph node dissection, ^†^ Mean (SD) is presented for descriptive orientation only and should be interpreted with caution, as the distribution of node counts is right-skewed and does not follow a normal distribution. The median and interquartile range are the appropriate summary statistics for these data. | | | |
