# Supplementary material for: Complications after sentinel node biopsy and inguinal lymph node dissection in penile cancer: a Danish national cohort study
Source: Int Urol Nephrol. 2026 Jun 11;58(11):4799–810. doi: 10.1007/s11255-026-05231-z (PMC13694304; doi:10.1007/s11255-026-05231-z)
Supplement: Supplementary file 2 [file 11255_2026_5231_MOESM2_ESM.docx]

**Supplementary table 2: All regression analyses**

Green: Highly protective factor (OR <0.85)

Red: High risk factor (OR >2)

Purple: Statistically significant (p<0.05)

**Supplementary Table 2. Complete regression analyses — predictors of postoperative complications after sentinel node biopsy and inguinal lymph node dissection.**

All analyses were performed using binary logistic regression with multiple imputation by chained equations (MICE, m=20 imputations) and pooling via Rubin's Rules. Univariable models tested each predictor individually; multivariable models included all predictors simultaneously. Multivariable analysis was not performed for outcomes with fewer than 10 events per variable (EPV <10): SNB lymphocele (~37 events, EPV ~4), ILND wound infection (~35 events, EPV ~3.5), and ILND lymphocele (~34 events, EPV ~3.4); these outcomes are reported with univariable results only.

For lymphoedema, two multivariable models are presented. Model 1 includes total nodes removed across all groins and total number of procedures as additional predictors alongside procedure group. Model 2 substitutes maximum unilateral nodes removed from a single groin in place of total nodes removed. Both models are presented to formally test whether nodal burden predicts lymphoedema independently of procedure type.

Predictors included in all primary and secondary models: age (continuous, per year), BMI (continuous, per kg/m²), smoking status (ever vs never), Charlson Comorbidity Index (continuous, per point), ASA classification (binary: I–II vs III–V), inguinal tumour at diagnosis (binary), histologically verified metastatic pelvic lymph nodes (binary), procedure-specific lymph node yield (continuous, per node), and metastatic nodes removed (binary and continuous, entered separately). Prior ipsilateral SNB was additionally included in all ILND models.

ASA: American Society of Anesthesiologists; BMI: body mass index; CCI: Charlson Comorbidity Index; CI: confidence interval; EPV: events per variable; ILND: inguinal lymph node dissection; OR: odds ratio; SNB: sentinel node biopsy.

| **Primary analyses** | | | | | | |
| --- | --- | --- | --- | --- | --- | --- |
| SNB CD ≥1: Univariable | | |  | SNB CD ≥1: Multivariable | | |
| **Predictor** | **OR (95% CI)** | **p-value** |  | **Predictor** | **OR (95% CI)** | **p-value** |
| Nodes removed, per node | 1.15 (1.01–1.31) | 0.030 |  | Age, per year | 1.00 (0.98-1.02) | 0.949 |
| ASA (III–V vs I–II) | 1.33 (0.90–1.96) | 0.150 |  | BMI, per kg/m² | 1.01 (0.97-1.05) | 0.611 |
| Smoking status (ever vs never) | 1.16 (0.78–1.73) | 0.450 |  | Smoking status (ever vs never)king status | 1.14 (0.76-1.70) | 0.528 |
| BMI, per kg/m² | 1.01 (0.97–1.05) | 0.594 |  | CCI | 1.00 (0.86-1.15) | 0.955 |
| Metastatic nodes removed, per node | 1.08 (0.78–1.50) | 0.643 |  | ASA | 1.31 (0.85-2.04) | 0.223 |
| Inguinal tumour at diagnosis | 1.12 (0.63–2.02) | 0.695 |  | Inguinal tumour at presentation | 1.10 (0.59-2.06) | 0.757 |
| CCI, per point | 1.02 (0.93–1.12) | 0.721 |  | Metastatic pelvic nodes, histologically verified | 0.70 (0.15-3.34) | 0.658 |
| Metastatic pelvic nodes, histologically verified | 0.78 (0.18–3.41) | 0.736 |  | Nodes removed, per node | 1.14 (1.01-1.30) | 0.039 |
| Age, per year, per year | 1.00 (0.99–1.02) | 0.886 |  | Metastatic nodes removed (binary, yes/no) | 0.94 (0.56-1.57) | 0.814 |
| Metastatic nodes removed (binary, yes/no) | 1.01 (0.62–1.65) | 0.966 |  |  | | |

| SNB CD ≥2: Univariable | | |  | SNB CD ≥2: Multivariable | | |
| --- | --- | --- | --- | --- | --- | --- |
| **Predictor** | **OR (95% CI)** | **p-value** |  | **Predictor** | **OR (95% CI)** | **p-value** |
| Nodes removed, per node | 1.17 (1.03-1.34 | 0.018 |  | Age, per year | 0.99 (0.97-1.02) | 0.481 |
| ASA (III–V vs I–II) | 1.25 (0.82-1.89 | 0.299 |  | BMI, per kg/m² | 1.00 (0.96-1.04) | 0.953 |
| Metastatic nodes removed (binary, yes/no) | 0.78 (0.44-1.38 | 0.397 |  | Smoking status (ever vs never) | 1.03 (0.68-1.57) | 0.888 |
| CCI, per point | 1.03 (0.94-1.14) | 0.505 |  | CCI, per point | 1.05 (0.91-1.21) | 0.490 |
| Metastatic pelvic nodes, histologically verified | 0.84 (0.20-3.55 | 0.814 |  | ASA (III–V vs I–II) | 1.20 (0.75-1.92) | 0.450 |
| Smoking status (ever vs never) | 1.04 (0.69-1.58 | 0.846 |  | Inguinal tumour at presentation | 0.98 (0.50-1.96) | 0.965 |
| Nodes removed, per node | 0.96 (0.66-1.41 | 0.852 |  | Metastatic pelvic nodes, histologically verified | 0.93 (0.21-4.22) | 0.929 |
| Inguinal tumour at presentation | 0.99 (0.52-1.90 | 0.973 |  | Nodes removed, per node | 1.18 (1.03-1.35) | 0.016 |
| BMI, per kg/m² | 1.00 (0.96-1.04 | 0.977 |  | Metastatic nodes removed (binary, yes/no) | 0.73 (0.41-1.32) | 0.304 |
| Age, per year | 1.00 (0.98-1.02 | 0.997 |  |  | | |

| ILND CD ≥1: Univariable | | |  | ILND CD ≥1: Multivariable | | |
| --- | --- | --- | --- | --- | --- | --- |
| **Predictor** | **OR (95% CI)** | **p-value** |  | **Predictor** | **OR (95% CI)** | **p-value** |
| Nodes removed, per node | 1.09 (1.00-1.18) | 0.046 |  | Age, per year | 1.00 (0.96-1.05) | 0.832 |
| CCI, per point | 1.14 (0.99-1.32) | 0.065 |  | BMI, per kg/m² | 1.05 (0.98-1.11) | 0.179 |
| Metastatic pelvic nodes, histologically verified | 2.30 (0.89-5.95) | 0.086 |  | Smoking status (ever vs never) | 1.22 (0.65-2.28) | 0.527 |
| Inguinal tumour at presentation | 1.40 (0.79-2.48) | 0.245 |  | CCI, per point | 1.23 (0.99-1.53) | 0.062 |
| Age, per year | 1.01 (0.99-1.04) | 0.333 |  | ASA (III–V vs I–II) | 0.63 (0.31-1.31) | 0.216 |
| Metastatic nodes removed, per node | 1.06 (0.93-1.20) | 0.370 |  | Inguinal tumour at presentation | 1.45 (0.68-3.09) | 0.339 |
| BMI, per kg/m² | 1.02 (0.97-1.07) | 0.497 |  | Metastatic pelvic nodes, histologically verified | 1.64 (0.53-5.08) | 0.387 |
| Prior ipsilateral SNB | 0.84 (0.48-1.48) | 0.545 |  | Nodes removed, per node | 1.10 (1.00-1.20) | 0.039 |
| Smoking status (ever vs never) | 1.17 (0.66-2.09) | 0.592 |  | Metastatic nodes removed (binary, yes/no) | 0.82 (0.36-1.87) | 0.642 |
| Metastatic nodes removed (binary, yes/no) | 1.11 (0.64-1.94) | 0.700 |  | Prior ipsilateral SNB | 0.96 (0.41-2.24) | 0.922 |
| ASA (III–V vs I–II) | 0.96 (0.54-1.71) | 0.892 |  |  | | |

| ILND CD ≥2: Univariable | | |  | ILND CD ≥2: Multivariable | | |
| --- | --- | --- | --- | --- | --- | --- |
| **Predictor** | **OR (95% CI)** | **p-value** |  | **Predictor** | **OR (95% CI)** | **p-value** |
| Nodes removed, per node | 1.11 (1.02-1.21) | 0.017 |  | Age, per year | 1.01 (0.97-1.05) | 0.746 |
| Metastatic pelvic nodes, histologically verified | 2.41 (0.93-6.22) | 0.070 |  | BMI, per kg/m² | 1.05 (0.98-1.13) | 0.131 |
| Inguinal tumour at presentation | 1.48 (0.81-2.70) | 0.196 |  | Smoking status (ever vs never) | 1.21 (0.63-2.34) | 0.568 |
| BMI, per kg/m² | 1.03 (0.98-1.09) | 0.218 |  | CCI, per point | 1.12 (0.89-1.40) | 0.324 |
| CCI, per point | 1.08 (0.93-1.25) | 0.289 |  | ASA (III–V vs I–II) | 0.83 (0.39-1.76) | 0.629 |
| Prior ipsilateral SNB | 0.86 (0.47-1.56) | 0.622 |  | Inguinal tumour at presentation | 1.71 (0.77-3.79) | 0.186 |
| Age, per year | 1.01 (0.98-1.03) | 0.695 |  | Metastatic pelvic nodes, histologically verified | 1.92 (0.65-5.67) | 0.235 |
| Metastatic nodes removed, per node | 1.03 (0.90-1.18) | 0.698 |  | Nodes removed, per node | 1.12 (1.02-1.23) | 0.015 |
| Smoking status (ever vs never) | 1.13 (0.61-2.08) | 0.699 |  | Metastatic nodes removed (binary, yes/no) | 0.58 (0.23-1.43) | 0.234 |
| ASA (III–V vs I–II) | 1.08 (0.59-1.98) | 0.799 |  | Prior ipsilateral SNB | 0.87 (0.35-2.18) | 0.771 |
| Metastatic nodes removed (binary, yes/no) | 0.96 (0.53-1.71) | 0.877 |  |  | | |

| **Secondary analyses** | | | | | | |
| --- | --- | --- | --- | --- | --- | --- |
| SNB Wound infection: Univariable | | |  | SNB Wound infection: Multivariable | | |
| **Predictor** | **OR (95% CI)** | **p-value** |  | **Predictor** | **OR (95% CI)** | **p-value** |
| Nodes removed, per node | 1.22 (1.06-1.41) | 0.007 |  | Age, per year | 0.98 (0.95-1.00) | 0.068 |
| Age, per year | 0.98 (0.96-1.00) | 0.055 |  | BMI, per kg/m² | 0.97 (0.92-1.02) | 0.229 |
| CCI, per point | 0.94 (0.84-1.06) | 0.315 |  | Smoking status (ever vs never) | 0.91 (0.56-1.48) | 0.700 |
| Metastatic nodes removed (binary, yes/no) | 0.74 (0.39-1.42) | 0.367 |  | CCI, per point | 1.02 (0.86-1.20) | 0.857 |
| Inguinal tumour at presentation | 1.33 (0.69-2.58) | 0.392 |  | ASA (III–V vs I–II) | 1.42 (0.83-2.45) | 0.204 |
| BMI, per kg/m² | 0.98 (0.93-1.03) | 0.440 |  | Inguinal tumour at presentation | 1.27 (0.62-2.60) | 0.509 |
| ASA (III–V vs I–II) | 1.18 (0.74-1.91) | 0.486 |  | Metastatic pelvic nodes, histologically verified | 1.28 (0.27-5.96) | 0.755 |
| Metastatic nodes removed, per node | 0.92 (0.59-1.43) | 0.701 |  | Nodes removed, per node | 1.22 (1.05-1.41) | 0.008 |
| Smoking status (ever vs never) | 0.94 (0.58-1.50) | 0.786 |  | Metastatic nodes removed (binary, yes/no) | 0.62 (0.31-1.24) | 0.180 |
| Metastatic pelvic nodes, histologically verified | 1.13 (0.27-4.77) | 0.871 |  |  | | |

| SNB Lymphocele: Univariable  *Note: ~37 events with 9 predictors (EPV ~4), multivariable not modelled | | |
| --- | --- | --- |
| **Predictor** | **OR (95% CI)** | **p-value** |
| Nodes removed, per node | 1.34 (1.11-1.62) | 0.002 |
| Smoking status (ever vs never) | 1.94 (0.93-4.05) | 0.077 |
| Age, per year | 1.02 (0.99-1.05) | 0.122 |
| ASA (III–V vs I–II) | 1.65 (0.85-3.22) | 0.140 |
| Metastatic nodes removed, per node | 1.34 (0.84-2.13) | 0.227 |
| CCI, per point | 1.09 (0.93-1.27) | 0.285 |
| Inguinal tumour at presentation | 0.48 (0.11-2.00) | 0.310 |
| BMI, per kg/m² | 1.01 (0.95-1.08) | 0.716 |
| Metastatic nodes removed (binary, yes/no) | 1.14 (0.49-2.63) | 0.761 |
| Metastatic pelvic nodes, histologically verified | 1.15 (0.15-8.70) | 0.895 |

| ILND Wound infection: Univariable  *Note: ~35 events with 10 predictors (EPV ~3.5), multivariable not modelled | | |
| --- | --- | --- |
| **Predictor** | **OR (95% CI)** | **p-value** |
| Nodes removed, per node | 1.10 (0.99-1.22) | 0.066 |
| Prior ipsilateral SNB | 0.59 (0.29-1.23) | 0.158 |
| ASA (III–V vs I–II) | 0.58 (0.26-1.32) | 0.193 |
| Metastatic pelvic nodes, histologically verified | 1.84 (0.64-5.27) | 0.256 |
| Inguinal tumour at presentation | 1.46 (0.70-3.03) | 0.312 |
| BMI, per kg/m² | 1.03 (0.97-1.10) | 0.324 |
| CCI, per point | 0.91 (0.75-1.10) | 0.327 |
| Age, per year | 0.98 (0.95-1.02) | 0.374 |
| Metastatic nodes removed (binary, yes/no) | 1.14 (0.55-2.35) | 0.717 |
| Smoking status (ever vs never) | 1.13 (0.53-2.43) | 0.747 |
| Metastatic nodes removed, per node | 1.02 (0.87-1.21) | 0.788 |

| ILND Lymphocele: Univariable  *Note: ~34 events with 10 predictors (EPV ~3.4), multivariable not modelled | | |
| --- | --- | --- |
| **Predictor** | **OR (95% CI)** | **p-value** |
| Metastatic pelvic nodes, histologically verified | 4.64 (1.80-11.94) | 0.002 |
| CCI, per point | 1.23 (1.03-1.47) | 0.021 |
| Inguinal tumour at presentation | 2.35 (1.12-4.91) | 0.023 |
| Age, per year | 1.03 (1.00-1.07) | 0.076 |
| Nodes removed, per node | 1.07 (0.97-1.19) | 0.186 |
| ASA (III–V vs I–II) | 0.85 (0.39-1.84) | 0.672 |
| Smoking status (ever vs never) | 0.86 (0.41-1.81) | 0.686 |
| BMI, per kg/m² | 0.99 (0.92-1.06) | 0.701 |
| Metastatic nodes removed, per node | 1.02 (0.86-1.21) | 0.843 |
| Metastatic nodes removed (binary, yes/no) | 0.93 (0.45-1.94) | 0.855 |
| Prior ipsilateral SNB | 0.98 (0.46-2.07) | 0.951 |

**Lymphoedema analyses (Patient level)**

**(Base model – Without ‘Max unilateral nodes removed, per node’, ‘Total nodes removed, per node’ and ‘Total number of procedures, per procedure’)**

| Lymphoedema: Univariable | | | | Lymphoedema: Multivariable | | |
| --- | --- | --- | --- | --- | --- | --- |
| **Predictor** | **OR (95% CI)** | **p-value** |  | **Predictor** | **OR (95% CI)** | **p-value** |
| Metastatic pelvic nodes, histologically verified | 4.09 (1.75-9.55) | 0.001 |  | Age, per year | 1.02 (0.99-1.05) | 0.147 |
| CCI, per point | 0.95 (0.86-1.05) | 0.299 |  | BMI, per kg/m² | 1.07 (1.03-1.12) | 0.002 |
| Age, per year | 1.00 (0.98-1.01) | 0.617 |  | Smoking status (ever vs never)1 | 0.89 (0.57-1.38) | 0.587 |
| Smoking status (ever vs never)1 | 0.93 (0.63-1.37) | 0.721 |  | CCI, per point | 0.91 (0.76-1.08) | 0.277 |
| ASA (III–V vs I–II) | 0.95 (0.63-1.44) | 0.817 |  | ASA (III–V vs I–II) | 0.82 (0.48-1.38) | 0.445 |
| BMI, per kg/m² | 1.06 (1.03-1.10) | <0.001 |  | Inguinal tumour at presentation | 1.56 (0.87-2.80) | 0.140 |
| Inguinal tumour at presentation | 3.17 (2.01-5.00) | <0.001 |  | Metastatic pelvic nodes, histologically verified | 1.59 (0.56-4.53) | 0.381 |
| Procedure group: ILND only | 9.83 (4.36-22.14) | <0.001 |  | Procedure group: ILND only | 6.84 (2.70-17.30) | <0.001 |
| Procedure group: SNB + ILND | 9.16 (5.93-14.16) | <0.001 |  | Procedure group: SNB + ILND | 8.78 (5.56-13.85) | <0.001 |

**(Second model – Including ‘Max unilateral nodes removed, per node’, ‘Total nodes removed, per node’ and ‘Total number of procedures, per procedure’)**

| Lymphoedema: univariable | | |  | Lymphoedema: Multivariable model 1 (total nodes removed) | | |
| --- | --- | --- | --- | --- | --- | --- |
| **Predictor** | **OR (95% CI)** | **p-value** |  | **Predictor** | **OR (95% CI)** | **p-value** |
| Procedure group: SNB + ILND | 9.16 (5.93-14.16) | <0.001 |  | Age, per year | 1.02 (0.99-1.05) | 0.139 |
| Max unilateral nodes removed, per node | 1.27 (1.20-1.34) | <0.001 |  | BMI, per kg/m² | 1.07 (1.02-1.12) | 0.002 |
| Total nodes removed, per node | 1.18 (1.13-1.22) | <0.001 |  | Smoking status (ever vs never)1 | 0.88 (0.57-1.38) | 0.586 |
| Total number of procedures, per procedure | 2.91 (2.13-3.99) | <0.001 |  | CCI, per point | 0.91 (0.77-1.08) | 0.290 |
| Procedure group: ILND only | 9.83 (4.36-22.14) | <0.001 |  | ASA (III–V vs I–II) | 0.82 (0.49-1.39) | 0.463 |
| Inguinal tumour at presentation | 3.18 (2.02-5.00) | <0.001 |  | Inguinal tumour at presentation | 1.51 (0.82-2.77) | 0.184 |
| BMI, per kg/m² | 1.06 (1.03-1.10) | <0.001 |  | Metastatic pelvic nodes, histologically verified | 1.53 (0.51-4.55) | 0.446 |
| Metastatic pelvic nodes, histologically verified | 3.94 (1.67-9.30) | 0.002 |  | Procedure group: ILND only | 5.27 (1.85-14.97) | 0.002 |
| CCI, per point | 0.95 (0.86-1.05) | 0.299 |  | Procedure group: SNB + ILND | 8.00 (3.89-16.47) | <0.001 |
| Age, per year | 1.00 (0.98-1.01) | 0.617 |  | Total nodes removed, per node | 1.03 (0.98-1.09) | 0.279 |
| Smoking status (ever vs never)1 | 0.93 (0.64-1.37) | 0.729 |  | Total number of procedures, per procedure | 0.87 (0.52-1.43) | 0.576 |
| ASA (III–V vs I–II) | 0.95 (0.63-1.44) | 0.817 |  |  |  |  |
|  | | |  | | | |
|  |  |  |  | Lymphoedema : Multivariable model 2 (max unilateral nodes removed) | | |
|  |  |  |  | **Predictor** | **OR (95% CI)** | **p-value** |
|  |  |  |  | Age, per year | 1.02 (0.99-1.05) | 0.120 |
|  |  |  |  | BMI, per kg/m² | 1.07 (1.02-1.12) | 0.002 |
|  |  |  |  | Smoking status (ever vs never)1 | 0.88 (0.57-1.37) | 0.572 |
|  |  |  |  | CCI, per point | 0.91 (0.76-1.08) | 0.260 |
|  |  |  |  | ASA (III–V vs I–II) | 0.83 (0.49-1.41) | 0.491 |
|  |  |  |  | Inguinal tumour at presentation | 1.54 (0.84-2.82) | 0.164 |
|  |  |  |  | Metastatic pelvic nodes, histologically verified | 1.55 (0.52-4.61) | 0.429 |
|  |  |  |  | Procedure group: ILND only | 5.29 (1.88-14.91) | 0.002 |
|  |  |  |  | Procedure group: SNB + ILND | 7.24 (3.26-16.07) | <0.001 |
|  |  |  |  | Max unilateral nodes removed, per node | 1.05 (0.97-1.14) | 0.265 |
|  |  |  |  | Total number of procedures, per procedure | 0.91 (0.56-1.47) | 0.700 |
